# Clinical Outcomes and Women’s Experiences before and after the Introduction of Mifepristone into Second-Trimester Medical Abortion Services in South Africa

**DOI:** 10.1371/journal.pone.0161843

**Published:** 2016-09-01

**Authors:** Deborah Constant, Jane Harries, Thokozile Malaba, Landon Myer, Malika Patel, Gregory Petro, Daniel Grossman

**Affiliations:** 1 Women’s Health Research Unit, School of Public Health and Family Medicine, University of Cape Town, Cape Town, South Africa; 2 Division of Epidemiology & Biostatistics, School of Public Health and Family Medicine, University of Cape Town, Cape Town, South Africa; 3 Department of Obstetrics & Gynaecology, University of Cape Town and Groote Schuur Hospital, Cape Town, South Africa; 4 Department of Obstetrics & Gynaecology, University of Cape Town and New Somerset Hospital, Cape Town, South Africa; 5 Ibis Reproductive Health, Oakland, California, United States of America; NHS lothian and University of Edinburgh, UNITED KINGDOM

## Abstract

**Objective:**

To document clinical outcomes and women’s experiences following the introduction of mifepristone into South African public sector second-trimester medical abortion services, and compare with historic cohorts receiving misoprostol-only.

**Methods:**

Repeated cross-sectional observational studies documented service delivery and experiences of women undergoing second-trimester medical abortion in public sector hospitals in the Western Cape, South Africa. Women recruited to the study in 2008 (n = 84) and 2010 (n = 58) received misoprostol only. Those recruited in 2014 (n = 208) received mifepristone and misoprostol. Consenting women were interviewed during hospitalization by study fieldworkers with respect to socio-demographic information, reproductive history, and their experiences with the abortion. Clinical details were extracted from medical charts following discharge. Telephone follow-up interviews to record delayed complications were conducted 2–4 weeks after discharge for the 2014 cohort.

**Results:**

The 2014 cohort received 200 mg mifepristone, which was self-administered 24–48 hours prior to admission. For all cohorts, following hospital admission, initial misoprostol doses were generally administered vaginally: 800 mcg in the 2014 cohort and 600 mcg in the earlier cohorts. Women received subsequent doses of misoprostol 400 mcg orally every 3–4 hours until fetal expulsion. Thereafter, uterine evacuation of placental tissue was performed as needed. With one exception, all women in all cohorts expelled the fetus. Median time-to-fetal expulsion was reduced to 8.0 hours from 14.5 hours (p<0.001) in the mifepristone compared to the 2010 misoprostol-only cohort (time of fetal expulsion was not recorded in 2008). Uterine evacuation of placental tissue using curettage or vacuum aspiration was more often performed (76% vs. 58%, p<0.001) for those receiving mifepristone; major complication rates were unchanged. Hospitalization duration and extreme pain levels were reduced (p<0.001), but side effects of medication were similar or more common for the mifepristone cohort. Overall satisfaction remained unchanged (95% vs. 91%), while other acceptability measures were higher (p<0.001) for the mifepristone compared to the misoprostol-only cohorts.

**Conclusion:**

The introduction of a combined mifepristone-misoprostol regimen into public sector second-trimester medical abortion services in South Africa has been successful with shorter time-to-abortion events, less extreme pain and greater acceptability for women. High rates of uterine evacuation for placental tissue need to be addressed.

## Introduction

Accessible and effective provision of second-trimester abortion is particularly important in settings where a high proportion of women seek abortion in the second trimester, as is common in low- and middle-income countries (LMICs) [1, 2]. South Africa’s abortion legislation of 1996 allows for abortion between 12 and 20 weeks for several indications, including on socio-economic grounds [3]. In the Western Cape Province of South Africa, 28% of all abortions are performed in the second trimester, which is higher than reported for the United States, United Kingdom, Nepal and the Russian Federation [4–8], although lower than in some parts of India [9, 10].

Both surgical and medical methods for second-trimester abortion are considered safe and effective when performed by skilled providers, and major complications are rare events [11–13]. However, shortages of physicians trained in dilation and evacuation (D&E) are a common problem, and medical methods using recommended regimens are increasingly used in resource-constrained settings [1, 2, 14, 15]. Randomized controlled trials (RCTs) comparing the combined mifepristone-misoprostol regimen to misoprostol used alone in the second trimester have consistently shown improvements in efficacy and time to abortion [14, 16–18]. Observational studies have documented similar outcomes following introduction of the combined regimen into services [19–24]. However, even in LMICs where mifepristone combined with misoprostol is standard of care for first-trimester abortion, this regimen has been less commonly used for later procedures. Possible reasons include limited recognition of its greater efficacy over the misoprostol-only regimen for second trimester procedures, and that comparatively less effort has been directed to advocating for the use of mifepristone than for first trimester procedures, as the procedure take places in hospital settings [14]. In addition, in South Africa, mifepristone is not registered for use in the second trimester, and it was only added to the national essential drug list for this indication in 2012.

Medical methods for abortion have been shown to be acceptable to women in many settings, both for first- and second-trimester procedures, as well as for managing incomplete abortion [11, 17, 25–28]. Factors reducing acceptability of first-trimester medical abortion with mifepristone include treatment failure, extreme pain and bleeding, inconvenience and anxiety [26–29]. In contrast, for second-trimester medical abortion, prolonged induction duration and high levels of pain reduce women’s acceptability and satisfaction with their procedure [17, 30].

With a shortage of physicians in the South African public sector who are skilled in D&E and willing to provide the service [31], medical abortion is generally the standard of care in the second trimester. In South Africa prior to 2013, second-trimester medical abortion was provided using a regimen of misoprostol only.

Previous research among women undergoing second-trimester abortion in South Africa using the misoprostol-only regimen reported safe abortion provision; however, the study reported delays in accessing care, and prolonged hospitalization beyond 2 days in many cases [11]. The mifepristone-misoprostol regimen for medical abortion was introduced into several public sector hospitals in the Western Cape Province in 2013 and 2014.

Implementation of new second-trimester abortion services into public sector teaching hospitals in LMICS s can be challenging as limited capacity, high patient-to-staff ratios, extensive referral areas, complicated referral pathways, high rates of staff rotation are commonplace [1, 7, 11, 32–34]. We aimed to add to the limited literature on second-trimester abortion from such settings by describing clinical outcomes and women’s experiences following the introduction of mifepristone into medical abortion services and comparing these to previous cohorts receiving misoprostol only.

## Methods

### Study design and participants

**Fig 1:**
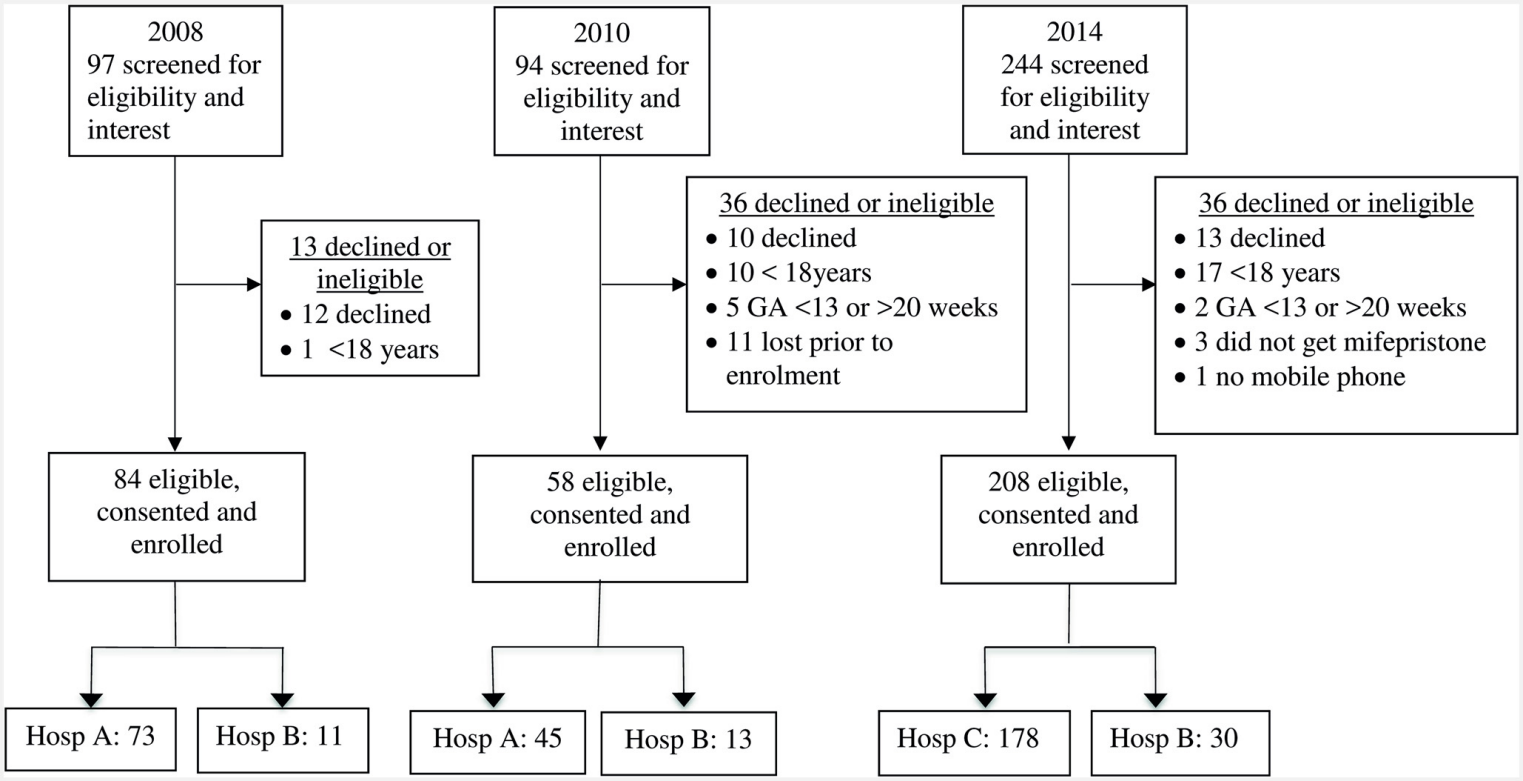
Participant recruitment to 2008, 2010 and 2014 cohorts.

The historic cohorts receiving misoprostol-only were recruited from February through July 2008 and April through August 2010 at Obstetric and Gynecology departments of two public sector teaching hospitals (Hospitals A & B, Fig 1) in the Western Cape providing medical abortion. Both hospitals are general specialist hospitals with 24-hour availability of surgical, anesthesia and blood transfusion services. Hospital A is in an urban and Hospital B in a semi-rural location, both with extensive referral areas. These two cohorts (2008 and 2010) were combined into a single misoprostol-only group for this analysis. The third cohort (referred to as the mifepristone group) received mifepristone for self-administration at home followed by admission for misoprostol and was recruited from October 2013 through June 2014, also at two public sector hospitals. This combined mifepristone-misoprostol regimen was introduced into a newly expanded service at Hospital C, an urban, highly specialized facility, and into Hospital B from the previous 2008/2010 cohorts, with Hospital A switching from medical abortion to a D&E service (Fig 1). In South Africa, second-trimester medical abortion takes place in secondary level hospitals. Hospitals A, B and C were the only facilities in the Western Cape providing the service. Additional hospitals in the province provide limited D&E services [11]. Ethical approval for all studies was given by the University of Cape Town Human Research Ethics Committee and the Allendale Investigational Review Board.

### Procedures

In all cohorts, women requesting abortion were referred from primary care facilities with a confirmed pregnancy and gestational age dating by ultrasound. At study facilities, women underwent further examination by physicians, received counselling and provided consent for the procedure, and a booking was arranged for admission to the general gynecology ward.

Study recruitment and procedures were similar for all cohorts. During the recruitment periods, within logistical constraints of one fieldworker per facility, all women undergoing medical abortion were approached for participation. Eligibility criteria included having been assessed by physicians and deemed medically appropriate for the service, having consented for medical abortion, being age 18 years or older, pregnant with a gestational age at time of mifepristone administration between 12 weeks 1 day and 20 weeks 0 days, and able to communicate in English or isiXhosa. Written informed consent to participate in the study was obtained following a confirmed booking for admission or on admission. Trained, experienced study fieldworkers not involved in service provision administered structured face-to-face interviews recording socio-demographic information, reproductive history, and women’s experience of the abortion procedure. Questions were standardized and identical for all cohorts. Interviews were conducted initially after admission and again prior to discharge or within 48 hours thereafter by phone. Investigators abstracted clinical information related to the abortion from hospital records after discharge. Fieldworkers conducted an additional follow-up telephone interview 2–4 weeks after discharge to collect information about delayed complications for the mifepristone group.

### Study outcomes

The primary outcome was 24-hour fetal expulsion rate (defined from first misoprostol dose to fetal expulsion; time of fetal expulsion was not recorded for the 2008 cohort). Other clinical outcomes included drug administration, time-to-fetal expulsion, rates of uterine evacuation for placental tissue and time-to-abortion completion (defined as time from first misoprostol dose to placental expulsion if no evacuation, or completion of evacuation, if done). In addition, hospitalization duration, analgesia, major complications (death, abdominal surgery, hospital readmission, hemorrhage requiring transfusion, infection treated with intravenous antibiotics and/or seizure) and post-abortion contraception were recorded. Women’s experiences included presence of side effects (yes/no), and level of pain, overall satisfaction (how would you describe your overall satisfaction with your abortion?) and acceptability (would you recommend the method to a friend?) measured on 5-point scales.

### Analysis

Data were analyzed using Stata v.13. Data for the 2008 and 2010 misoprostol-only cohorts were combined to improve statistical power of the historical data. Missing data were not imputed, and only valid percentages were reported. Descriptive statistics were reported for participant characteristics and study outcomes. Five point scales were collapsed to three categories for analysis, and groups were compared using Chi-squared tests for proportions or Kruskal-Wallis tests for medians. Kaplan-Meier analyses and log-rank tests were used to compare unadjusted time-to-fetal expulsion between mifepristone and misoprostol-only groups. To adjust for confounding and differences in drug administration, hazard ratios (HRs) for time-to-expulsion were calculated for exposure to mifepristone, adjusted for significant covariate differences identified *a priori*, (age, gestational age, prior vaginal delivery, parity, prior abortion) and stratified by quartiles of total misoprostol dosage. HRs were also calculated for the majority subgroup of participants receiving their first misoprostol dose via the vaginal route of administration (PV).

### Sample size

The desired sample size was based on fetal expulsion rate, as the more direct measure of abortion effectiveness compared to abortion completion, which is dependent on physician practice with regard to curettage. Reported fetal expulsion rates within 24 hours range between 93–97% for the mifepristone-misoprostol regimen [13, 15] and for our misoprostol-only 2010 cohort was 72% (95% CI: 63%– 81%). To be conservative, we based our sample size calculation on a 90% fetal expulsion rate within 24 hours for the mifepristone-misoprostol group and 81% for the misoprostol-only group (the upper limit of the 2010 cohort 95% CI). We calculated that a sample size of 131 participants in the mifepristone cohort would suffice to detect a significant difference in fetal expulsion rate within 24 hours between the mifepristone and the misoprostol-only cohorts using a one-sided 2.5% level test of significance, with a power of 90%. To allow for analysis of covariate effects, we planned to include 200 women in the mifepristone cohort.

## Results

For the 2008 and 2010 misoprostol-only and mifepristone cohorts respectively, 86% (84/97), 62% (58/94) and 85% (208/244) of women screened for participation were enrolled into the study. Overall, enrolled women constituted 50% (350/700) of all women undergoing medical abortion at study facilities during the study periods. Participant enrolment for all cohorts, including reasons for non-participation are shown in Fig 1.

### Participant characteristics

Significant differences in participant characteristics between the 2008 and 2010 misoprostol-only cohorts were gestational age at initiation of abortion (median (interquartile range): 18.1 weeks (17.6–19.9) and 16.5 weeks (14.9–17.6) respectively; and the proportion reporting prior abortions (7% [5/74] in 2008 compared to none in 2010). Participant characteristics for the combined 2008/2010 misoprostol group and the mifepristone group are shown in Table 1. There were no significant differences between the combined misoprostol-only group and the mifepristone group for age, education, formal housing, paid work, home language, parity or prior vaginal delivery. More participants receiving mifepristone reported prior abortions and median gestational age on admission was 1 week later than the misoprostol-only group (Table 1).

**Table 1:** Participant characteristics by medication regimen.

Characteristic	2008/2010 misoprostol-only	2014 mifepristone-misoprostol	p-value*
Age (years)	n = 142	n = 208	
18–25	77 (54%)	94 (45%)	0.373
26–35	53 (37%)	92 (44%)	
>35	12 (8%)	22 (11%)	
High school education	n = 129^‡^	n = 208	
Completed Grade 12	62 (48%)	98 (47%)	0.477
Home language	n = 138^#^	n = 208	
Xhosa	86 (62%)	151 (73%)	0.118
English	26 (19%)	22 (11%)	
Afrikaans	17 (12%)	25 (12%)	
Other	9 (7%)	10 (5%)	
Employment	n = 129^‡^	n = 208	
Paid work	50 (39%)	80 (39%)	0.523
Parity	n = 142	n = 208	
Nulliparous	26 (18%)	41 (20%)	0.428
Prior vaginal delivery	n = 129^‡^	n = 208	
	95 (74%)	144 (69%)	0.229
Prior abortion	n = 129^‡^	n = 208	
	5 (4%)	21 (10%)	0.027
Gestational age (weeks)**	n = 142	n = 208	
12.1–16.0	36 (25%)	30 (14%)	<0.001
16.1–18.0	54 (38%)	55 (26%)	
18.1–20.0	52 (37%)	123 (59%)	

*p-value for chi squared tests of differences between misoprostol-only and mifepristone-misoprostol groups.

^‡^13 records with missing data

^#^4 records with missing data.

**Gestational age at abortion commencement.

Date of first misoprostol for 2008 and 2010 cohorts, and date of mifepristone for 2014 cohort.

### Drug Administration

All women in the mifepristone cohort were instructed to self-administer 200 mg mifepristone orally at home, 24–48 hours prior to their booked admission date. Women with gestations within 1–2 days of the legal limits for medical abortion were given priority bookings, and admission was arranged in order to manage bed availability. Only 1 participant was admitted directly following assessment for mifepristone administration in the hospital, as her travel time home and back would have been excessive. The median and inter-quartile range (IQR) for the mifepristone-misoprostol interval was 49 hours (IQR 4–53). Intervals were <48 hours for 44% (91/208) participants and >72 hours for 2% (4/208). All participants reported they took the mifepristone at home, and none aborted prior to hospital admission.

Following admission, administration of misoprostol was incorporated into the general ward routine. Misoprostol doses and administration routes are shown in Table 2. Although there was an attempt to standardize the protocol, dosage regimens sometimes differed across facilities, or according to physician preference in certain situations, such as with prior caesarean section. Generally, misoprostol-alone cohorts received an initial dose of 600 mcg, with 21% (28/135) receiving 400 or 200 mcg. Administration routes were mostly vaginal (PV) (84%, 113/135), with a minority receiving either oral or sublingual administration. Subsequent dosing was 400 mcg at 3- or 4-hourly intervals. If no expulsion had occurred after 13 doses, following a rest period, misoprostol was started again. If expulsion did not occur, other prostaglandins were used. For the combined mifepristone-misoprostol regimen the first misoprostol dose was generally 800 mcg administered PV, with 24% (49/208) receiving either 400 mcg orally or 600 mcg PV or by the sublingual route. Subsequent dosing was mostly 400 mcg orally, with 7% (13/208) sublingually, up to 10 doses if needed, sometimes followed by a rest period. The median total dose of misoprostol and the median number of misoprostol doses were less in the mifepristone cohort (1600 mcg vs. 1800 mcg; 2 vs. 3, p<0.001).

**Table 2:** Misoprostol dosage regimen.

Misoprostol regimen	2008/2010 misoprostol-only	2014 mifepristone-misoprostol
Dosage of 1st misoprostol	n = 135^‡^	n = 208
200 mcg	2 (1.5%)	0 (0%)
400 mcg	26 (19.3%)	13 (6.3%)
600 mcg	107 (79.3%)	36 (17.3%)
800 mcg	0 (0%)	159 (76.4%)
Route of administration of 1st dose	n = 135^‡^	n = 208
Vaginal	113 (83.7%)	178 (85.6%)
Oral	20 (14.8%)	13 (6.3%)
7Sublingual	2 (1.5%)	17 (8.2%)
Dosage of 2nd misoprostol	n = 133^‡^	n = 191
200 mcg	11 (8.3%)	0 (0%)
400 mcg	120 (90.2%)	179 (93.7%)
600 mcg	2 (1.5%)	12 (6.3%)
800 mcg	0 (0%)	0 (0%)
Route of administration of 2nd dose	n = 133^‡^	n = 191
Vaginal	1 (0.8%)	0 (0%)
Oral	130 (97.7%)	178 (93.2%)
Sublingual	2 (1.5%)	13 (6.8%)
Total misoprostol dosage (mcg) Median (IQR)*	1800 (1400–2400)	1600 (1200–2000)
Number of misoprostol doses Median (IQR)*	4 (4–6)	3 (2–4)

* p<0.001. Kruskal-Wallis test for median differences between misoprostol-only and mifepristone-misoprostol groups.

^‡^7 records with missing data.

### Clinical outcomes

**Fig 2:**
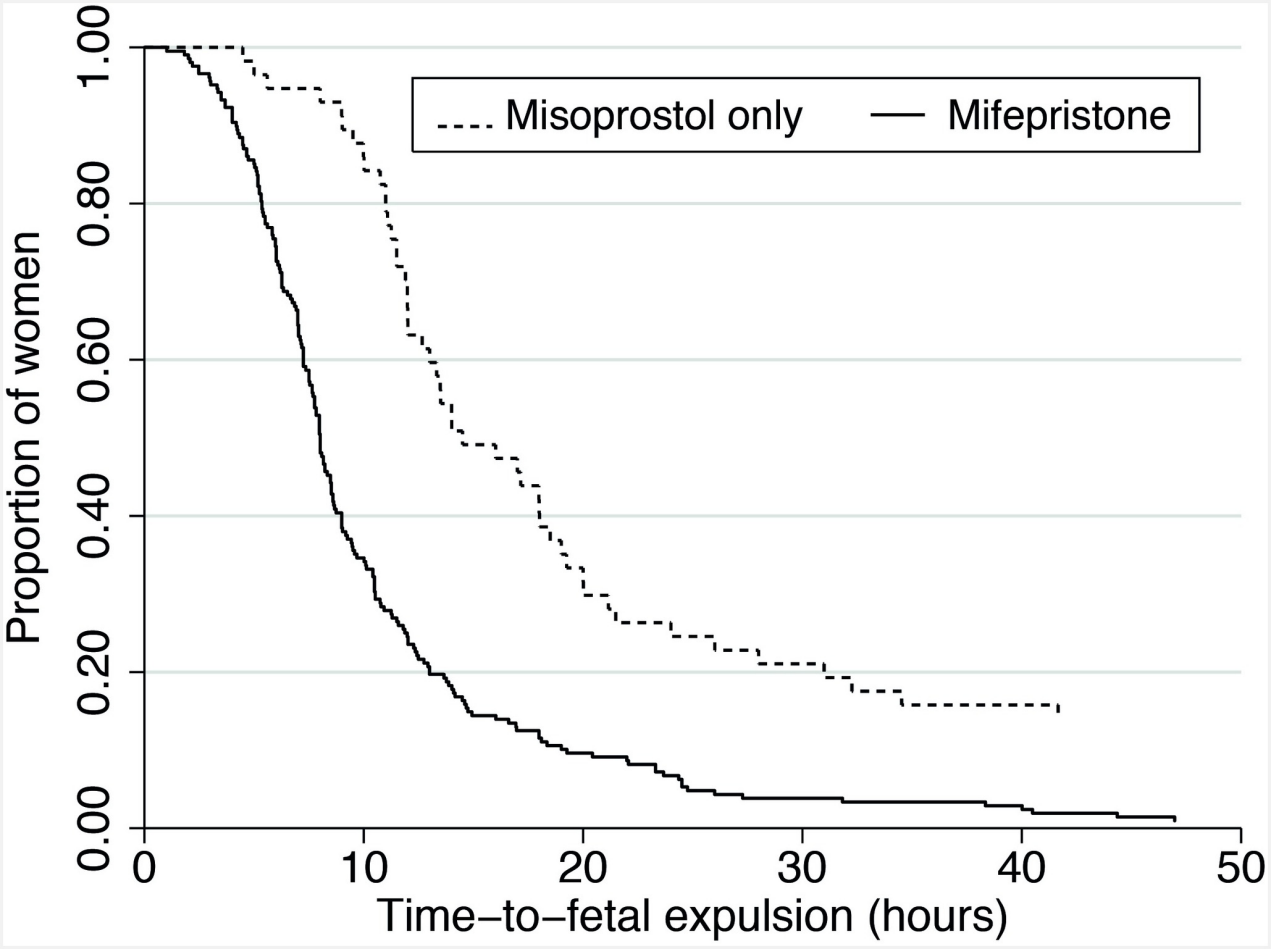
Time-to-fetal expulsion (unadjusted) for 2010 misoprostol-only (n = 57) and mifepristone groups (n = 208).

Fetal expulsion occurred in all participants except one in the 2008 misoprostol-only cohort, who was transferred to another facility for D&E. Median time-to-fetal expulsion was shorter (8.0 vs. 14.5 hours; p<0.001), and the proportion with fetal expulsion within 24 hours higher (93% [194/208] vs. 77% [44/57]; p<0.001) for the mifepristone cohort compared to the misoprostol-only 2010 cohort. Kaplan-Meier survival curves and log rank tests for unadjusted time-to-fetal expulsion demonstrated significantly shortened intervals for the mifepristone cohort (p<0.001, Fig 2). Hazard ratios for fetal expulsion from Cox proportional models adjusted for gestational age, prior abortion and prior vaginal delivery and stratified by quartiles of total misoprostol dose were significantly higher for the mifepristone group, overall and for the PV subgroup (p<0.05 for all strata, S1 Table).

Uterine evacuation of placental tissue was performed according to physicians’ judgement, and there was higher rate of uterine evacuation of placental tissue among the mifepristone group over the misoprostol-only group (76% [159/208] vs. 58% [82/142]; p<0.001, Table 3). Methods used were curettage in the 2008/2010 cohorts and vacuum aspiration or curettage for the mifepristone group. Despite higher evacuation rates, median time-to-abortion completion, which includes time to completion of uterine evacuation, if performed, was significantly shorter in the mifepristone group (11.1 vs. 24.8 hours; p<0.001) compared to misoprostol only and a significantly higher proportion had completed their abortion within 24 hours (88% [177/208] vs. 46% [62/134]; p<0.001, Table 3).

**Table 3:** Procedure details and clinical outcomes.

Clinical outcomes	2008/2010 misoprostol-only	2014 mifepristone-misoprostol	p-value*
Time from 1^st^ dose misoprostol to fetal expulsion	n = 57^‡^	n = 208	<0.001
Median (IQR) (hours)	14.5 (11.5–24.0)^‡^	8.0 (6.0–11.9)	<0.001
Fetal expulsion <24 hours	n = 57^‡^	n = 208	
n (%)	44 (77%)^‡^	194 (93%)	<0.001
Uterine evacuation performed	n = 142	n = 208	
n (%)	82 (58%)	159 (76%)	<0.001
Time from 1^st^ dose of misoprostol to abortion completion**	n = 134^#^	n = 208	<0.001
Median (IQR) (hours)	24.8 (17.8–37.6)	11.1 (8.7–17.0)	
Complete abortion <24 hours	n = 134^#^	n = 208	<0.001
n (%)	62 (46%)	177 (88%)	
Hospitalization	n = 142	n = 208	
Same day discharge	1 (1%)	0 (0%)	<0.001
1 night	62 (43%)	161 (77%)	
≥2 nights	80 (56%)	47 (23%)	
Analgesia given	n = 142	n = 208	
n (%)	63 (44%)	173 (83%)	<0.001
Major complications	n = 142	n = 208	
n (%)	11 (8%)	12 (6%)	0.791
Haemorrhage requiring transfusion	8 (6%)	9 (4%)	
Infection treated with IV antibiotics	3 (2%)	2 (1%)	
Possible seizure	0 (0%)	1 (<1%)	
Received post-abortion family planning	n = 127***	n = 206 ^§^	
n (%)	125 (98%)	204 (99%)	0.623
Injectable	111 (87%)	139 (67%)	
Oral contraceptives	9 (7%)	4 (2%)	
Intrauterine device	5 (4%)	19 (9%)	
Implant	0 (0%)	42 (20%)	

*p-value of Chi-squared tests for differences between misoprostol-only and mifepristone-misoprostol groups.

^‡^Data only recorded for 2010 cohort, 1 record with missing data.

**Abortion completion defined as either placental expulsion if no surgery or surgery.

^#^8 records with missing data,

^§^ 2 records with missing data,

*** 15 records with missing data

Duration of hospitalization was significantly shorter for the mifepristone group (77% [161/208] vs. 44% [62/142] staying one night or less; p<0.001). The rate of major complications was similar for the two groups (6% [12/208] vs. 8% [11/142]; p = 0.791). Complications included hemorrhage requiring blood transfusion, infection treated with IV antibiotics and possible seizure (Table 3). All these complications were identified during hospitalization except for one in the mifepristone cohort, which was identified at the follow-up call. More women received analgesia in the mifepristone compared to the misoprostol groups (83% [173/208] vs. 44% [63/142]; p<0.001). Almost all participants in both mifepristone and misoprostol-only groups (99% [204/208] and 98% 125/127]; p = 0.623) received a family planning method post-abortion. The most common method in both groups was the injectable (67% [139/204] vs. 87% [111/125]), with a shift towards the implant (introduced into the public sector in 2013) in the mifepristone group (20% [42/204]).

### Women’s experiences

More participants in the mifepristone group experienced nausea (55% [112/205] vs. 22% [29/129]; p<0.001), vomiting (45% [92/205] vs. 27% [35/129]; p<0.001) and tiredness (76% [155/203] vs. 56% [72/129]; p<0.001). Other symptoms experienced similarly by both mifepristone and misoprostol-only groups, respectively, were diarrhea (58% [119/205] vs. 62% [80/129]; p = 0.472), dizziness (41% [85/205] vs. 40% [52/129]; p = 0.835) and headache (35% [72/203] vs. 37%, [48/129]; p = 0.441). There was no significant linear association between pain levels and groups (Table 4); however, fewer women in the mifepristone compared to the misoprostol group experienced extreme pain compared to other levels of pain (13% [26/205 vs. 41% [53/129]; p<0.001). Trends in overall satisfaction were similar for both group, while more reported they would recommend the abortion method to a friend in the mifepristone group (Table 4; p<0.001).

**Table 4:** Women’s experiences of the abortion.

	2008/2010 misoprostol-only	2014 mifepristone-misoprostol	p-value*
Overall pain during abortion experience	n = 129 ^‡^	n = 205^#^	
Extreme pain	53 (41%)	26 (13%)	0.320
High pain	23 (18%)	108 (53%)	
Moderate pain	19 (15%)	64 (31%)	
Slight pain	14 (11%)	18 (8%)	
No pain	20 (16%)	2 (1%)	
Overall satisfaction with abortion	n = 129 ^‡^	n = 205^#^	
Very or somewhat satisfied	117 (91%)	195 (95%)	0.127
Neutral	8 (6%)	3 (2%)	
Somewhat or very dissatisfied	4 (3%)	7 (3%)	
Would recommend the abortion method to a friend who needed one at same gestational age	n = 129 ^‡^	n = 205^#^	
Highly or somewhat agree	90 (70%)	183 (89%)	<0.001
Neutral	4 (3%)	1 (0.5%)	
Somewhat or highly disagree	35 (7%)	21 (10%)	

*p-value for chi squared test for trend of linear association between groups and levels of outcome

^‡^13 records with missing,

^#^3 records with missing data.

## Discussion

Compared to the previous misoprostol-only regimen, the new service regimen reduced time-to-abortion events and hospitalization. The 93% fetal expulsion rate within 24 hours in our mifepristone group is slightly lower compared to the 94–98% reported in RCTs and case series [15, 16, 22–24]. It is possible that the variability of the mifepristone-misoprostol interval in our study (range; 27–77 hours) contributed to the marginally lower expulsion rate compared to these studies. In addition, the dosing and route of administration of misoprostol in our 2014 mifepristone group was not wholly consistent with the WHO evidence-based clinical guidelines for mifepristone in combination with misoprostol, which advise 800 mcg vaginally, followed by 400 mcg vaginally or sublingual, q 3 hrs., and 400mcg vaginally or sublingual, repeated q 3 hrs. for misoprostol only [35]. This may also have contributed to the slightly lower 24-hour expulsion rates than reported elsewhere. Deviations from WHO protocol were due to physician preference in cases with prior caesarean section, and the use of the oral route for the second misoprostol dose is in accordance with Royal College of Obstetricians and Gynaecologists (RCOG) guidance, which has been followed in other settings [23].

Other research has reported much lower rates of placental retention for second-trimester medical abortion with mifepristone [16], and our finding of a higher surgical evacuation rate with the mifepristone regimen is likely to be specific to the service delivery setting. In both study groups time-to-abortion completion may have been extended due to lack of operating theatre availability when uterine evacuation was performed, which was facility-dependent. The high rate of evacuation, especially in the mifepristone group, may be related to the introduction of the new service in teaching facilities with a high rotation of junior doctors. This may have also contributed to the shorter time-to-abortion completion in this group. Routine use of evacuation varies across settings and institutions [13, 36]; however, it is acknowledged that clinical experience in assessment of abortion completion and manual assistance with placental expulsion is needed to avoid this practice [13]. Further training of health professionals in this regard has been implemented in our study facilities.

The introduction of self-administration of mifepristone at home prior to admission for second-trimester medical abortion allowed for planning for admission while eliminating additional hospital visits for mifepristone where admission was delayed by more than 48 hours. Although women’s convenience was not specifically catered to in this study, flexible timing of admission without requiring mifepristone to be taken on-site gives women time to arrange their personal affairs, if needed, and we consider it a beneficial service delivery option. Most women stayed at least one night in hospital as incorporation of abortion care into the general ward routine delayed women receiving their misoprostol while this study was in progress. To facilitate same-day discharge, a day ward administering misoprostol immediately at early morning admission is recommended as the most cost-effective model of service provision for medical abortion [13, 36]. This was considered feasible in China and Europe [15, 23, 37], and was recently implemented at hospital C (personal communication, MP, 2015). It is expected that the new regimen may reduce costs per woman served, which we plan to explore in a future analysis.

The number of women experiencing hemorrhage requiring transfusion was similar in both the mifepristone and misoprostol-only groups and (Table 3). The proportion in the mifepristone group (4.3%) was comparable to the 4% rate reported previously for all deliveries in a South African hospital in 2010 [38], but higher than other studies on second-trimester medical abortion, which vary from 0.7–3% [16, 23, 24]. The transfusion threshold is context- and provider-dependent, and most women having transfusions had a documented drop in hemoglobin with signs and/or symptoms of anemia. Possible reasons for this higher rate of transfusion could be a high rate of baseline anemia, or the high HIV prevalence; nonetheless, interventions to reduce transfusion should be explored in future research. For other major complications, of the 5 cases with infections requiring IV antibiotics, 4 were detected during admission. While seemingly of different origin, the 1% infection rate in this study is comparable to that (8/1002) reported elsewhere at 2-week follow-up [23].

In terms of women’s experiences, both the mifepristone and the misoprostol-only groups reported a high prevalence of common side effects. The greater proportion in the mifepristone group experiencing nausea, vomiting and tiredness may be due to the higher initial dose of misoprostol, or possibly to mifepristone. In contrast, this group also reported less extreme pain levels and more received analgesia than the misoprostol-only cohorts; however, pain levels in all groups remained high, and more research on pain management is needed to better guide practice. The improved acceptability in the mifepristone group, despite the higher proportion who underwent curettage for retained placental tissue, is encouraging. Reduced hospitalization and less extreme pain may have contributed to a better experience. However, unmeasured differences between facilities and over time may also have played a part, and we cannot make conclusive statements in this regard from our data.

Limitations of this study are that the majority of participants in the mifepristone cohort groups were from a different facility from the historic cohorts due to changes in service delivery. Study groups were non-randomized. For example, gestational age was different in the two groups; however, we adjusted for known confounders, including gestational age, in the proportional hazards analysis (S1 Table). The comparison for time-to-fetal expulsion may have been underpowered due to the missing data in the 2008 cohort; nonetheless our study findings were statistically and clinically significant. The participant numbers were unbalanced between the two centers in all cohorts as Hospital B is a smaller site; however, proportions relative to the overall caseload for each center were consistent over the three phases of data collection. In addition, the study extended over six years of service provision, during which unmeasured confounding factors may have been influenced our findings. We did not conduct follow-up interviews for either of the misoprostol-only cohorts; however, the chart reviews were done subsequent to discharge, thus it is unlikely that we have underestimated major complications for this group. There may have been some social desirability bias, however this would not have differed between the study groups. The initial misoprostol dosages differed between the study groups, but they were generally consistent with WHO guidance, which recommends a lower dose for the misoprostol-only second-trimester regimen [35]. To take this into account, we stratified the proportional hazards analysis according to categories of overall misoprostol dosage (S1 Table). The misoprostol dosages sometimes differed from recommended regimens; however, in this observational study we aimed to observe real-world outcomes after implementing this new service. Finally, our findings are specific to the South African context where second-trimester medical abortion is performed in secondary level hospitals and may not be generalizable to other settings.

### Conclusions

This study reports clinical and acceptability improvements during introduction of a mifepristone-misoprostol regimen for medical abortion at 12–20 weeks gestational age at busy public sector hospitals in South Africa. Specific aspects that were successful included self-administration at home of mifepristone which allowed for admission planning, shortened procedure times, reduced hospitalization which increased the capacity of facilities to serve more women compared to previously, and increased acceptability.

## Supporting Information

S1 File 2008 2010 2014 Data file. (XLSX)

S1 Table Hazard ratios for mifepristone-misoprostol compared to misoprostol only stratified by misoprostol dose and adjusted for gestational age at abortion commencement, prior vaginal delivery and prior abortion. (DOCX)

## References

[1] Edelman A, Alemayehu T, Gebrehiwot Y, Kidenemariam S, Getachew Y. Addressing unmet need by expanding access to safe second trimester medical abortion services in Ethiopia, 2010–2014. Int J Gynaecol Obstet. 2015;128(2):177–8. 10.1016/j.ijgo.2014.07.035 25597962

[2] Baldwin M, Basnett I, Dangol D, Karki C, Castleman L, Edelman A. Notes from the field: expanding abortion services into the second trimester of pregnancy in Nepal (2007–2012). Contraception. 2014;90(6):562–4. 10.1016/j.contraception.2014.08.010 25266926

[3] Choice on Termination of Pregnancy, Act No.92 of 1996. Government Gazette, State Paper of the Republic of South Africa, (1996).

[4] Sinjani/TOP’s. 2014–2015 financial year. Western Cape Government: Health. [Online]. Available at: https://sinjani.pgwc.gov.za/live/sinjani (Restricted access). Western Cape Department of Health, South Africa. 2014.

[5] Berer M. A Critical Appraisal of Laws on Second Trimester Abortion. Reprod Health Matters. 2008;16(31, Suppl):3–13. 18772078 10.1016/S0968-8080(08)31395-0

[6] Harris LH. Second Trimester Abortion Provision: Breaking the Silence and Changing the Discourse. Reprod Health Matters. 2008;16(31, Suppl):74–81. 18772087 10.1016/S0968-8080(08)31396-2

[7] Samandari G, Wolf M, Basnett I, Hyman A, Andersen K. Implementation of legal abortion in Nepal: a model for rapid scale-up of high-quality care. Reprod Health. 2012;9(7):1742–4755. 10.1186/1742-4755-9-7 PMC3373381 22475782

[8] Zhirova IA, Frolova OG, Astakhova TM, Ketting E. Abortion-related maternal mortality in the Russian Federation. Stud Fam Plann. 2004;35(3):178–88. 15511061 10.1111/j.1728-4465.2004.00021.x

[9] Dalvie SS. Second trimester abortions in India. Reprod Health Matters. 2008;16(31):37–45. 10.1016/S0968-8080(08)31384-6 18772082

[10] Zavier AJ, Jejeebhoy S, Kalyanwala S. Factors associated with second trimester abortion in rural Maharashtra and Rajasthan, India. Glob Public Health. 2012;7(8):897–908. 10.1080/17441692.2011.651734 22263668

[11] Grossman D, Constant D, Lince N, Alblas M, Blanchard K, Harries J. Surgical and medical second trimester abortion in South Africa: A cross-sectional study. BMC Health Serv Res. 2011;11(1):1. 21929811 10.1186/1472-6963-11-224 PMC3196698

[12] Grossman D, Blanchard K, Blumenthal P. Complications after Second Trimester Surgical and Medical Abortion. Reprod Health Matters. 2008;16(31, Suppl):173–82. 18772098 10.1016/S0968-8080(08)31379-2

[13] Gemzell-Danielsson K, Lalitkumar S. Second Trimester Medical Abortion with Mifepristone–Misoprostol and Misoprostol Alone: A Review of Methods and Management. Reprod Health Matters. 2008;16(31, Suppl):162–72. 10.1016/S0968-8080(08)31371-8 18772097

[14] Dabash R, Chelli H, Hajri S, Shochet T, Raghavan S, Winikoff B. A double-blind randomized controlled trial of mifepristone or placebo before buccal misoprostol for abortion at 14–21 weeks of pregnancy. Int J Gynecol Obstet. 2015;130(1):40–4. 10.1016/j.ijgo.2015.02.023 25896965

[15] Mentula M, Suhonen S, Heikinheimo O. One- and two-day dosing intervals between mifepristone and misoprostol in second trimester medical termination of pregnancy—a randomized trial. Hum Reprod 2011;26(10):2690–7. 10.1093/humrep/der218 21798991

[16] Kapp N, Borgatta L, Stubblefield P, Vragovic O, Moreno N. Mifepristone in second-trimester medical abortion: a randomized controlled trial. Obstet Gynecol. 2007;110(6):1304–10. 18055725 10.1097/01.AOG.0000289577.32274.a5

[17] Ngoc NT, Shochet T, Raghavan S, Blum J, Nga NT, Minh NT, et al Mifepristone and misoprostol compared with misoprostol alone for second-trimester abortion: a randomized controlled trial. Obstet Gynecol. 2011;118(3):601–8. 10.1097/AOG.0b013e318227214e 21860289

[18] ACOG Practice Bulletin No. 135: Second-trimester abortion. Obstet Gynecol. 2013;121(6):1394–406. 10.1097/01.AOG.0000431056.79334.cc 23812485

[19] Kulkarni KK. Pre-induction with Mifepristone for Second Trimester Termination of Pregnancy. J Obstet Gynaecol India. 2014;64(2):102–4. 10.1007/s13224-013-0472-5 24757336 PMC3984646

[20] Nagaria T, Sirmor N. Misoprostol vs mifepristone and misoprostol in second trimester termination of pregnancy. J Obstet Gynaecol India. 2011;61(6):659–62. 10.1007/s13224-011-0118-4 23204686 PMC3307930

[21] Dickinson JE, Brownell P, McGinnis K, Nathan EA. Mifepristone and second trimester pregnancy termination for fetal abnormality in Western Australia: Worth the effort. Austr N Z J Obstet Gynaecol. 2010;50(1):60–4. 10.1111/j.1479-828X.2009.01117.x 20218999

[22] Goh SE, Thong KJ. Induction of second trimester abortion (12–20 weeks) with mifepristone and misoprostol: a review of 386 consecutive cases. Contraception. 2006;73(5):516–9. 16627037 10.1016/j.contraception.2005.12.004

[23] Ashok PW, Templeton A, Wagaarachchi PT, Flett GMM. Midtrimester medical termination of pregnancy: a review of 1002 consecutive cases. Contraception. 2004;69(1):51–8. 14720621 10.1016/j.contraception.2003.09.006

[24] Rose SB, Shand C, Simmons A. Mifepristone- and misoprostol-induced mid-trimester termination of pregnancy: a review of 272 cases. Austr N Z J Obstet Gynaecol. 2006;46(6):479–85. 10.1111/j.1479-828X.2006.00646.x 17116051

[25] Kopp Kallner H, Gomperts R, Salomonsson E, Johansson M, Marions L, Gemzell-Danielsson K. The efficacy, safety and acceptability of medical termination of pregnancy provided by standard care by doctors or by nurse-midwives: a randomised controlled equivalence trial. Br J Obstet Gynaecol. 2015;122(4):510–7. 10.1111/1471-0528.12982 25040643

[26] Kopp Kallner H, Fiala C, Gemzell-Danielsson K. Assessment of significant factors affecting acceptability of home administration of misoprostol for medical abortion. Contraception. 2012;85(4):394–7. 10.1016/j.contraception.2011.08.009 22067756

[27] Paul M, Iyengar K, Essen B, Gemzell-Danielsson K, Iyengar SD, Bring J, et al Acceptability of Home-Assessment Post Medical Abortion and Medical Abortion in a Low-Resource Setting in Rajasthan, India. Secondary Outcome Analysis of a Non-Inferiority Randomized Controlled Trial. PloS One. 2015;10(9):e0133354 10.1371/journal.pone.0133354 26327217 PMC4556554

[28] Cleeve A, Byamugisha J, Gemzell-Danielsson K, Mbona Tumwesigye N, Atuhairwe S, Faxelid E, et al Women's Acceptability of Misoprostol Treatment for Incomplete Abortion by Midwives and Physicians—Secondary Outcome Analysis from a Randomized Controlled Equivalence Trial at District Level in Uganda. PLoS One. 2016;11(2):e0149172 10.1371/journal.pone.0149172 26872219 PMC4752492

[29] Ngo TD, Park MH, Shakur H, Free C. Comparative effectiveness, safety and acceptability of medical abortion at home and in a clinic: a systematic review. Bull World Health Organ. 2011;89(5):360–70. 10.2471/BLT.10.084046 21556304 PMC3089386

[30] Andersson IM, Gemzell-Danielsson K, Christensson K. Caring for women undergoing second-trimester medical termination of pregnancy. Contraception. 2014;89(5):460–5. 10.1016/j.contraception.2014.01.012 24594432

[31] Harries J, Cooper D, Strebel A, Colvin CJ. Conscientious objection and its impact on abortion service provision in South Africa: a qualitative study. Reprod Health. 2014;11(1):16-4755-11-16. 10.1186/1742-4755-11-16 PMC3996040 24571633

[32] Harries J, Lince N, Constant D, Hargey A, Grossman D. The challenges of offering public second trimester abortion services in South Africa: Health providers' perspectives. J Biosoc Sci. 2012;44(02):197–208. 22088440 10.1017/S0021932011000678

[33] Harris LH, Grossman D. Confronting the challenge of unsafe second-trimester abortion. Int J Gynaecol Obstet. 2011;115(1):77–9. 10.1016/j.ijgo.2011.05.018 21820115

[34] Hoang Td T, Phan T, Huynh Nk T. Second Trimester Abortion in Viet Nam: Changing to Recommended Methods and Improving Service Delivery. Reprod Health Matters. 2008;16(31):145–50. 10.1016/S0968-8080(08)31393-7 18772095

[35] WHO. Clinical practice handbook for Safe Abortion. Geneva,Switzerland: World Health Organization 2014. 24624482

[36] Lalitkumar S, Bygdeman M, Gemzell-Danielsson K. Mid-trimester induced abortion: a review. Hum Reprod Update. 2007;13(1):37–52. 17050523 10.1093/humupd/dml049

[37] Hou S, Zhang L, Chen Q, Fang A, Cheng L. One- and two-day mifepristone-misoprostol intervals for second trimester termination of pregnancy between 13 and 16 weeks of gestation. Int J Gynaecol Obstet. 2010;111(2):126–30. 10.1016/j.ijgo.2010.06.008 20705290

[38] Basu J, Hartford L, Nzama N, Dayal C, Naidoo B, Chuene M, et al The use of blood transfusions in the obstetric unit of an academic hospital in South Africa. Southern Afr J Epidemiol Infect. 2012;27(1):34–6.

