# Supplementary material for: Clinical Outcomes and Women’s Experiences before and after the Introduction of Mifepristone into Second-Trimester Medical Abortion Services in South Africa
Source: PLoS One. 2016 Sep 1;11(9):e0161843. doi: 10.1371/journal.pone.0161843 (PMC5008795; doi:10.1371/journal.pone.0161843)
Supplement: S1 Table — (DOCX) [file pone.0161843.s002.docx]

**S1 Table. Hazard ratios for mifepristone-misoprostol compared to misoprostol only stratified by misoprostol dose and adjusted for gestational age at abortion commencement, prior vaginal delivery and prior abortion.**

|  | **Time-to-fetal expulsion** | |
| --- | --- | --- |
|  | All (n=263^†^) | PV route 1^st^ misoprostol dose (n=221) |
| Misoprostol dose (mcg) | HR (95%CI) | HR (95%CI) |
| 600-1200 | n=89 | n=59 |
|  | 3.1 (1.7-5.3) | 3.6 (1.4-9.3) |
|  | p<0.001 | p=0.008 |
| 1400-1600 | n=78 | n=77 |
|  | 10.8 (3.9-29.7) | 29.6 (6.4-136.9) |
|  | p<0.001 | p<0.001 |
| 1800-2200 | n=43 | n=40 |
|  | 6.8 (2.4-19.0) | 10 (2.2-48.2 |
|  | p<0.001 | p=0.003 |
| 2400-14600 | n=53 | n=45 |
|  | 3.8 (1.6-8.9) | 3.0 (1.2-7.7) |
|  | p=0.002 | p=0.021 |

^†^Data only recorded for 2010 cohort; 3 records with missing data for at least 1 variable in the model.
